# Supplementary material for: A meta-model of low back pain to examine collective expert knowledge of treatment effects and their mechanisms
Source: Eur Spine J. 2026 May 19;35(7):3795–808. doi: 10.1007/s00586-026-09932-y (PMC13372931; doi:10.1007/s00586-026-09932-y)
Supplement: Supplementary file 1 — Supplementary Material 1 [file 586_2026_9932_MOESM1_ESM.pdf]

A meta-model of low back pain to examine collective expert knowledge of treatment effects and their mechanisms

Online Resource 1: Participant Demographics.

Participant	Discipline	Country	Gender
1	Basic Science	Canada	F
2	Basic Science	USA	F
3	Basic Science	USA	M
4	Biomechanics	USA	M
5	Biomechanics	USA	M
6	Biomechanics	Netherlands	M
7	Chiropractic	UK	M
8	Chiropractic	USA	F
9	Chiropractic	Denmark	M
10	Chiropractic	Canada	M
11	Epidemiology	Australia	M
12	Epidemiology	Netherlands	M
13	Exercise Science	USA	M
14	Physical Medicine and Rehabilitation	USA	F
15	Physical Medicine and Rehabilitation	Netherlands	M
16	Physiotherapy/Neuroscience	Australia	M
17	Physiotherapy	USA	M
18	Physiotherapy	Belgium	M
19	Physiotherapy	USA	F
20	Physiotherapy	Australia	F
21	Physiotherapy	USA	M
22	Physiotherapy	Australia	F
23	Physiotherapy	Canada	F
24	Physiotherapy	Australia	M
25	Physiotherapy	USA	F
26	Psychology	Netherlands	M
27	Psychology	USA	F
28	Spine Surgery	Germany	M
29	Spine Surgery	USA	M
