# Supplementary material for: A meta-model of low back pain to examine collective expert knowledge of treatment effects and their mechanisms
Source: Eur Spine J. 2026 May 19;35(7):3795–808. doi: 10.1007/s00586-026-09932-y (PMC13372931; doi:10.1007/s00586-026-09932-y)
Supplement: Supplementary file 2 — Supplementary Material 2 [file 586_2026_9932_MOESM2_ESM.pdf]

A meta-model of low back pain to examine collective expert knowledge of treatment effects and their mechanisms

Online Resource 2: Changes to Component terminology and Domain classification since previous publication [2].

	Change
1	In consultation with the participant, “Counseling and Education about the aerobic exercise” was incorporated into “Cognitive behavioral therapy” and removed as a stand-alone Treatment/Intervention Component.
2	“Spinal manipulation” and “Manual therapy” were merged into a new Treatment/Intervention Component “Spinal manipulation/Manual therapy.”
3	“Alternative therapy” was incorporated into “Complementary treatments” and removed as a stand-alone Treatment/Intervention Component.
4	“Good clinical interventions (e.g., bio-medication physiotherapy psychosocial interventions)” was moved from “Pain relieving intervention” to “Multidisciplinary treatments (biopsychosocial treatments).”
5	Two Components “Patient's adherence to care” and “Environmental demands” proposed in the previous study, were removed, because there were no Connections with any other Components identified.
6	“Person-centered care” was reclassified from “Treatment/Intervention” to “Social/Work/Contextual” Domain.
7	In consultation with the participant, “Conservative musculoskeletal therapies such as manipulation massage and acupuncture” that was previously attributed to “Complementary Treatments” was moved to “Physical treatment.”
8	“Placebo” from a contributor that was previously attributed to “Psychological intervention” in Treatment/Intervention Domain was moved to “Positive psychological factors” in the Psychological factors Domain.
9	"Myofascial trigger point" was reclassified from “Treatment/Intervention” to “Tissue injury” Domain.
