# Supplementary figures and images for: A meta-model of low back pain to examine collective expert knowledge of treatment effects and their mechanisms
Source: Eur Spine J. 2026 May 19;35(7):3795–808. doi: 10.1007/s00586-026-09932-y (PMC13372931; doi:10.1007/s00586-026-09932-y)

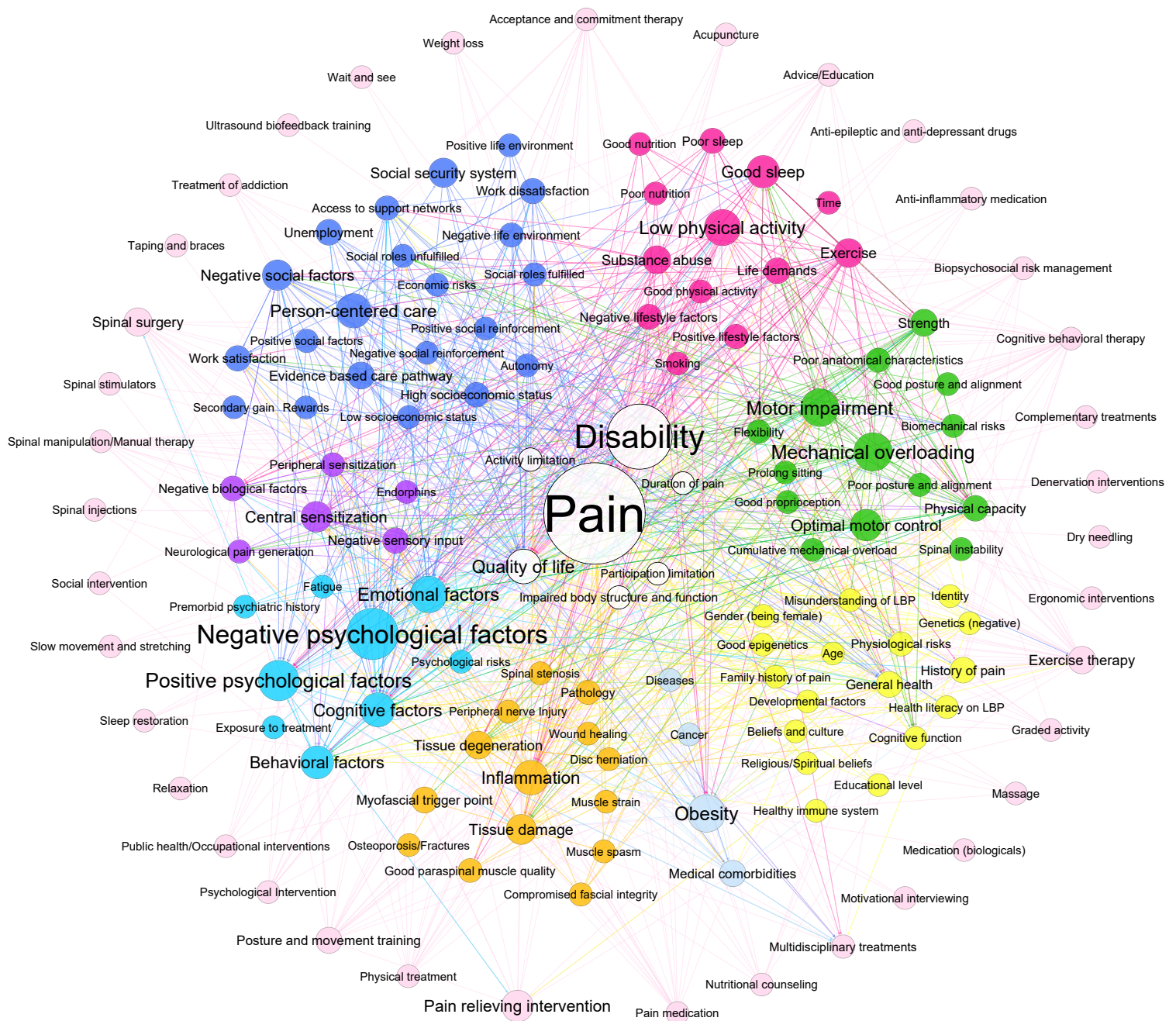

Supplement: Supplementary file 3 — Supplementary Material 3 [file 586_2026_9932_MOESM3_ESM.pdf]
